# An analysis of aging-related genes derived from the Genotype-Tissue Expression project (GTEx)

**DOI:** 10.1038/s41420-018-0093-y

**Published:** 2018-08-20

**Authors:** Kaiwen Jia, Chunmei Cui, Yuanxu Gao, Yuan Zhou, Qinghua Cui

**Affiliations:** 1 Department of Biomedical Informatics, Department of Physiology and Pathophysiology, Center for Noncoding RNA Medicine, MOE Key Lab of Cardiovascular Sciences, School of Basic Medical Sciences, Peking University, 38 Xueyuan Road, 100191 Beijing, China; 2 Center of Bioinformatics, Key Laboratory for Neuro-Information of Ministry of Education, School of Life Science and Technology, University of Electronic Science and Technology of China, 610054 Chengdu, China

**Keywords:** Ageing, Transcriptomics

## Abstract

Aging is a complex biological process that is far from being completely understood. Analyzing transcriptional differences across age might help uncover genetic bases of aging. In this study, 1573 differentially expressed genes, related to chronological age, from the Genotype-Tissue Expression (GTEx) project, were categorized as upregulated age-associated genes (UAGs) and downregulated age-associated genes (DAGs). Characteristics in evolution, expression, function and molecular networks were comprehensively described and compared for UAGs, DAGs and other genes. Analyses revealed that UAGs are more clustered, more quickly evolving, more tissue specific and have accumulated more single-nucleotide polymorphisms (SNPs) and disease genes than DAGs. DAGs were found with a lower evolutionary rate, higher expression level, greater homologous gene number, smaller phyletic age and earlier expression in body development. UAGs are more likely to be located in the extracellular region and to occur in both immune-relevant processes and cancer-related pathways. By contrast, DAGs are more likely to be located intracellularly and to be enriched in catabolic and metabolic processes. Moreover, DAGs are also critical in a protein–protein interaction (PPI) network, whereas UAGs have more influence on a signaling network. This study highlights characteristics of the aging transcriptional landscape in a healthy population, which may benefit future studies on the aging process and provide a broader horizon for age-dependent precision medicine.

## Introduction

Aging is considered to be a dominating risk factor for many fatal diseases, including cancer, cardiovascular diseases and neurodegenerative diseases^1–6^. A large number of studies have found that aging is associated with telomere attrition, mitochondrial dysfunction, DNA damage, immune system impairment etc., and can be inhibited by calorie restriction^7–11^. However, the detailed mechanisms involved in aging remain unclear. In recent years, rapidly developing high-throughput omics have provided a broader insight, with the identification of a number of longevity-relevant loci based on genome-wide association studies(GWAS) and epigenome analyses^12,13^. As previous studies have shown, aging is distinct at molecular, cellular and tissue levels^14^, which indicates that the relatively dynamic transcriptome might also provide important clues for the study of aging. A large number of human age-associated genes have been identified in previous transcriptomic studies, based on specific tissues like muscle, blood, skin, adipose, brain etc., and have been compared across tissues^15–24^. The identified age-associated genes in these studies vary from each other, which could be partly due to differences in the health condition of donors, sample size, sample quality, tissue, platform and the method of identifying age-associated genes. Also, the divergent results may be accounted for by the generally low repeatability of microarray data that most of the analyses were based on. In addition, given that aging is likely to confound with other factors, profiling the aging factor alone can be difficult, which may lead to biases in screening age-associated genes. Among these studies, the age-associated gene set from the Genotype-Tissue Expression (GTEx) project^25^ is of high quality. This set of aging-associated genes was screened out by using a regression model on large-sample RNA-Seq data, collected from >40 tissues from hundreds of healthy individuals; whereas sex, race, and tissue were controlled as a covariate to avoid biases^24^. However, characteristics of these genes still remain unexplored. A comprehensive analysis of these genes might help to improve the understanding of aging process and provide valuable clues for strategies in anti-aging interventions. Here, we comprehensively explored characteristics of the age-associated genes derived from GTEx. The results revealed that differences and interactions exist in evolution, expression, function, associated diseases and molecular network between the upregulated age-associated genes (UAGs) and downregulated age-associated genes (DAGs).

## Result

### The human transcriptional age-associated genes from GTEx

**Fig. 1:**
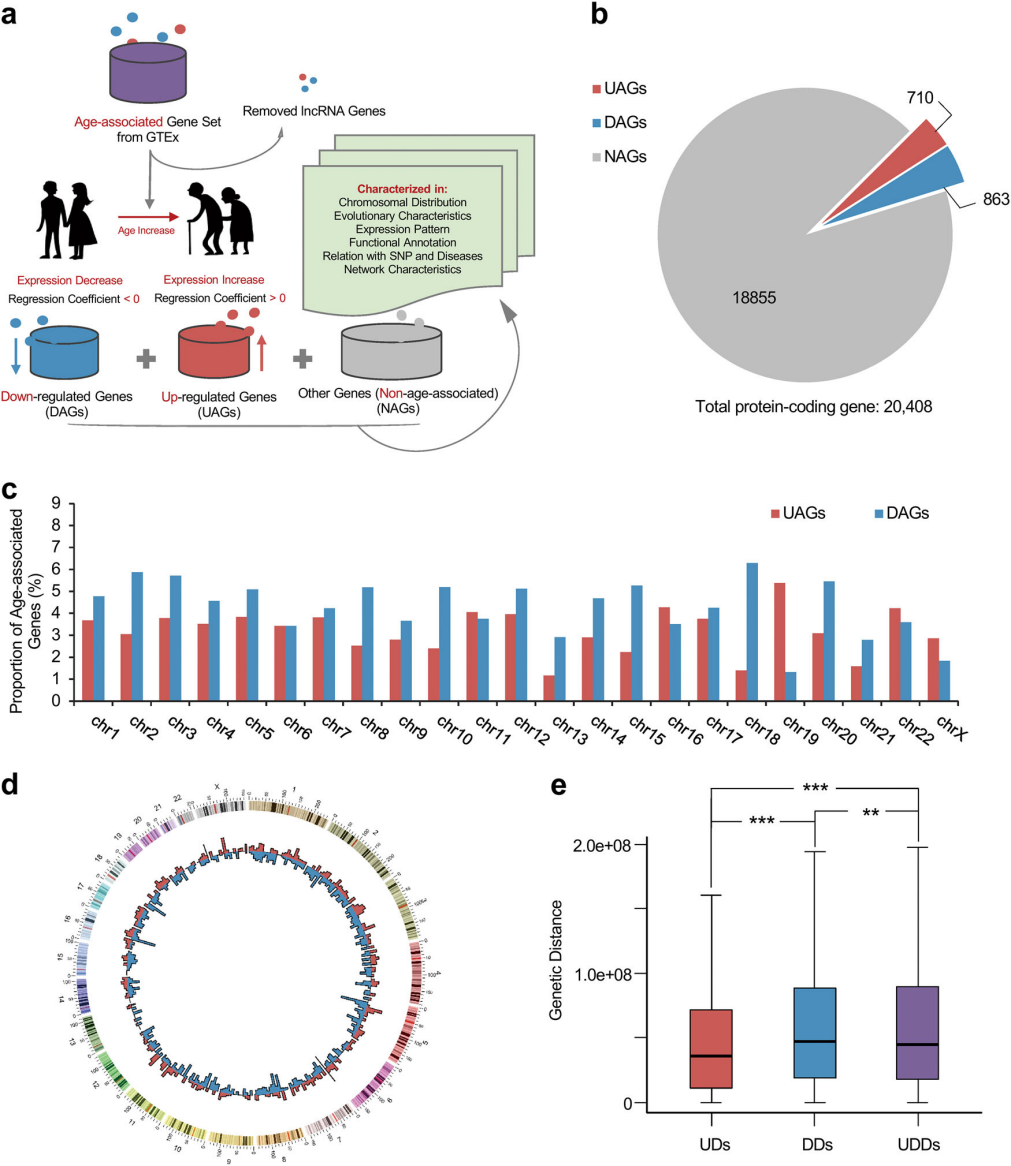
The transcriptional age-associated genes. **a** The workflow for characterizing age-associated genes. **b** A pie chart showing the proportion of age-associated genes within all protein-coding genes. **c** Chromosomal distribution of the 1573 age-associated genes. **d** Circos^54^ plot displaying the age-associated genes. **e** Genomic distances between age-associated genes. UDs distances of each pair of UAGs on the same chromosome, DDs distances of each pair of DAGs on the same chromosome, UDDs distances of any pair of genes on the same chromosome between UAGs and DAGs groups. **P < 0.01, ***P < 0.001 from Wilcoxon test

To characterize the age-associated genes, we extracted a protein-coding transcriptional age-associated gene set from a GTEx transcriptional analysis (see Materials and methods section). The gene set analysis procedure is summarized in Fig. 1a. Ultimately, we extracted 710 UAGs and 863 DAGs across tissues. The age-associated genes account for 7.71% of the protein-coding genes (Fig. 1b and Supplementary Table 1). Genomic information of UAGs and DAGs is shown in Supplementary Tables 2 and 3, respectively.

### Distribution of the age-associated genes across chromosomes

To characterize the age-associated genes at the genomic level, we investigated the chromosomal distribution pattern of the age-associated genes. Results showed age-associated genes are widespread across chromosomes. Over 97% of the age-associated genes are located on autosomes, where the proportion of DAGs is higher than that of UAGs, with the exception of five autosomes. Although no age-associated genes were found on the Y chromosome, possibly due to the adjustment for sex in the regression model used to discover age-associated genes, some UAGs (2.86%) and a smaller number of DAGs (1.83%) were observed on the X chromosome (Figs. 1c, d).

Genes with similar functions are likely to locate adjacently on chromosomes^26^. To study whether the age-associated genes are close to each other on chromosomes, we calculated the genomic distances of each pair of genes on the same chromosome, within one age-associated gene group only (UDs, DDs), and between groups (UDDs). Results showed these three distances are significantly different from each other (median values of UDs, DDs and UDDs are 3.52e + 7, 4.67e + 7 and 4.42e + 7, respectively). Of the three distances, UDs possess the smallest value compared with DDs (*P* = 7.52e-56, Wilcoxon test) and UDDs (*P* = 1.17e-57, Wilcoxon test). Surprisingly, UDDs show smaller value than DDs (*P* = 0.0038, Wilcoxon test) (Fig. 1e). These results suggest that on the same chromosome, UAGs are more clustered than DAGs and non-age-associated genes (NAGs); DAGs on the other hand are relatively dissociated, whereas some of them tend to be more adjacent to UAGs than other DAGs.

### Evolutionary characteristics and expression profile of age-associated genes

**Fig. 2:**
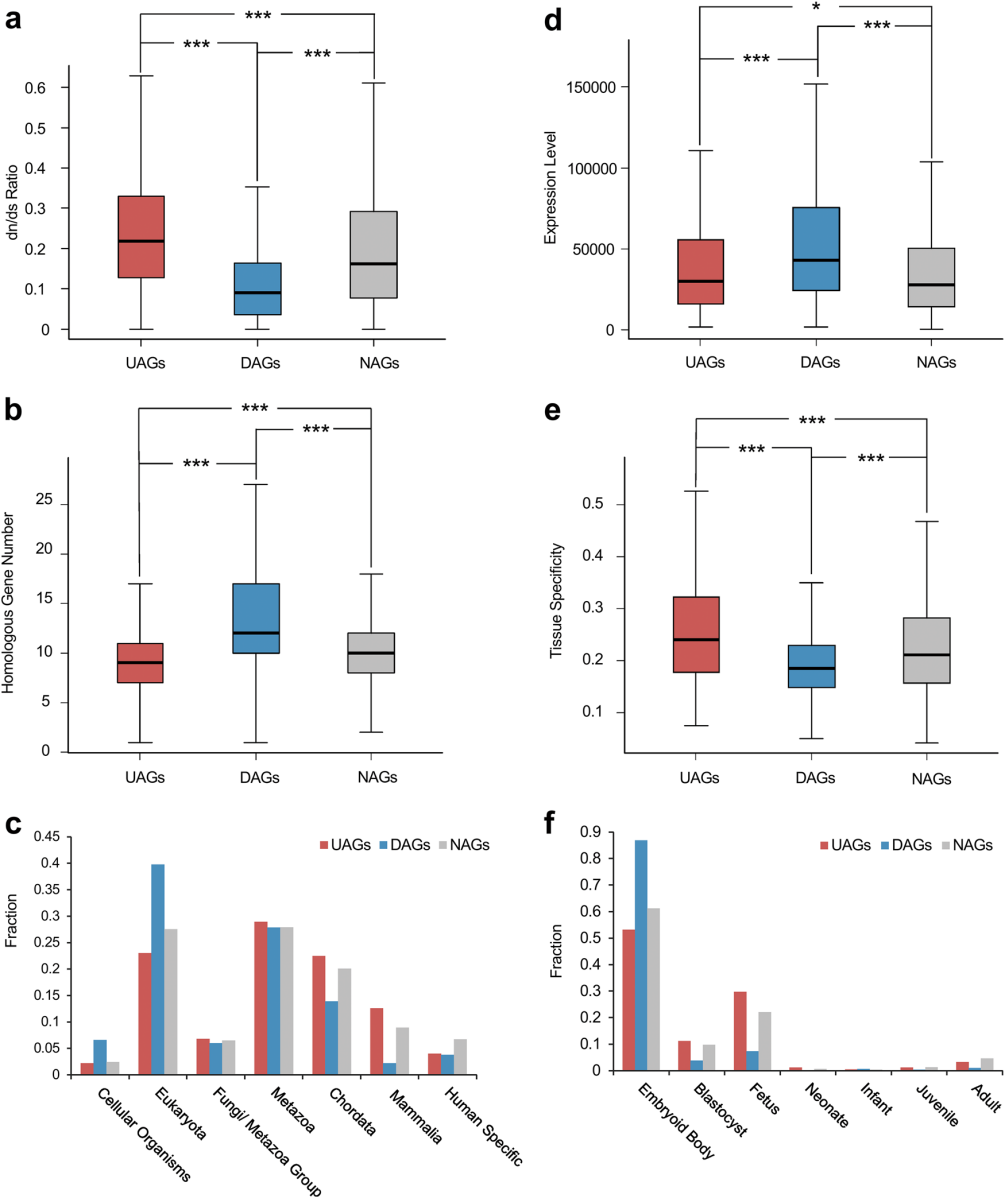
Evolutionary characteristic and expression profile of age-associated genes. Box plots and bar graphs show UAGs, DAGs and NAGs compared in the following characteristics: **a** dN/dS ratio of each human–mouse homolog gene. **b** Number of homologous genes. **c** Origin in phyletic evolution. **d** Average expression level across tissues. **e** Tissue specificity of gene expression. **f** Earliest expression stage. *P < 0.05, ***P < 0.001 from Wilcoxon test

To gain a better insight into the evolutionary background of genes, the evolutionary characteristics of age-associated genes were investigated in three aspects: evolutionary rate, homologous gene number and phyletic age. Results showed that DAGs have the lowest dN/dS ratio (*P* = 1.43e-50, Wilcoxon test), the highest homologous gene number (*P* = 5.73e-55 Wilcoxon test) and have a relatively earlier origin in phyletic age compared with UAGs (*P* = 7.37e-24, chi-squared test). The corresponding values for NAGs are *P* = 5.02e-45, *P* = 3.39e-60 and *P* = 4.74e-32. Comparatively, UAGs evolves most rapidly, with the smallest homologous gene number, and originated later than DAGs but earlier than NAGs (Figs. 2a–c; Table 1). These results revealed that DAGs are more conserved in evolution, suggesting the DAGs are more crucial to fundamental functions in humans. However, UAGs are less stable and have a shorter history, which implies they are likely to function in more specific and advanced functions.

**Table 1:** Statistical results for characteristic analyses in evolution and expression

	DAGs versus UAGs	DAGs versus NAGs	UAGs versus NAGs
dN/dS			
Median	U: 0.22	D: 0.09	N: 0.16
W-value	227,110	2,243,300	3,179,700
* P*-value	1.43e-50	5.02e-45	5.11e-12
Homologous gene number			
Median	U: 9	D: 12	N: 10
W-value	155,150	9,421,800	5,009,300
* P*-value	5.73e-55	3.39e-60	7.05e-9
Phyletic evolution			
Chi-square	121.64	160.46	24.01
* P*-value	7.37e-24	4.74e-32	5.21e-4
Average expression level			
Median	U: 29,873.5	D: 42,838	N: 27,993
W-value	196,500	7,431,900	4,885,500
* P*-value	2.91e-13	2.41e-37	0.032
Tissue specificity			
Median	U: 0.24	D: 0.19	N: 0.21
W-value	349,390	4,727,100	5,389,400
* P*-value	2.27e-31	2.73e-20	1.24e-11
Earliest expression stage			
Chi-square	149.99	164.91	19.266
* P*-value	7.77e-30	5.39e-33	0.0037
Essential genes			
Proportion	U: 11.83% (84 of 710)	D: 15.30% (132 of 863)	N: 11.92% (2248 of 18,855)
Chi-square	3.67	8.53	2.23e-4
* P*-value	0.06	3.49e-3	0.99

dN/dS ratio, homologous gene number and expression pattern were analyzed by Wilcoxon test, the phyletic evolution, earliest expression stage and essential genes enrichment analyses were performed by chi-squared test

Essential genes are those that function in basic biological processes and must exist for an organism to survive^27^. As expected, DAGs are significantly more enriched in human essential genes (15.3% of DAG genes), compared with NAGs (11.8%; *P* = 0.0035, chi-squared test) (Table 1). UAGs (11.8%) and NAGs (11.9%) have no significant difference.

The expression profile also provides important characteristics for a gene and often gives valuable clues to potential gene function. Therefore, we explored the expression characteristics of age-associated genes from three aspects: average expression level across tissue, tissue specificity and the earliest expression stage. We found DAGs have the highest expression level and lowest tissue specificity compared with UAGs and NAGs, suggesting DAGs have a more global function, providing basic support to the whole body. UAGs are significantly higher in expression level and tissue specificity than NAGs, which suggests UAGs are also important in function, but more tissue specific (Figs. 2d, e; Table 1). As for the earliest expression stage, although the stage of embryoid body is predominant for expression in all three groups, DAGs are far more expressed than UAGs and NAGs at this stage. UAGs on the other hand outnumber DAGs and NAGs in the following stages of blastocyst and fetus (Fig. 2f; Table 1). Altogether the age-associated genes tend to express in earlier stages than other genes, underlining they are likely to play important roles in early development.

### Functional annotation for age-associated genes

**Fig. 3:**
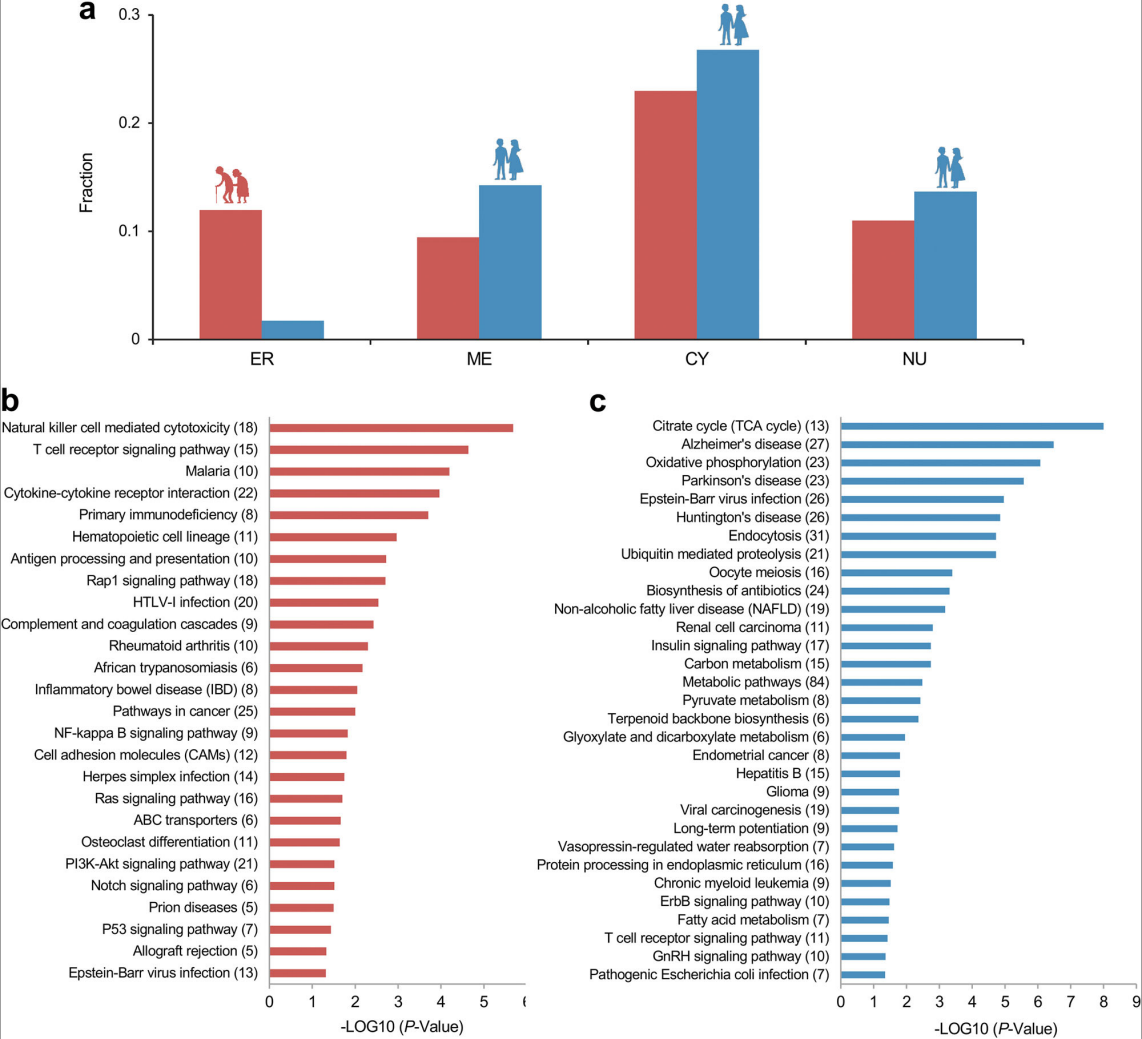
Functional enrichment analysis for age-associated genes. **a** The number of UAGs and DAGs in four subcellular locations is shown. ER extracellular region, ME membrane, CY cytoplasm, NU nucleus. UAGs and DAGs are presented in red and blue, respectively. **b** KEGG enrichment analysis for UAGs with the threshold of *P* < 0.05. **c** KEGG enrichment analysis for DAGs with the threshold of *P* < 0.05

Functional enrichment analyses were carried out to explore the functions of UAGs and DAGs. First, we investigated the subcellular locations of UAGs and DAGs to see if they are located differently. Genes located in nucleus (NU), cytoplasm (CY), membrane (ME) and extracellular region (ER) were filtered from gene ontology (GO) terms and the age-associated genes were mapped into these cellular locations. The number of UAGs and DAGs in these four locations were, 78 versus 118 (10.99% versus 13.67%, NU), 163 versus 231 (22.96% versus 26.77%, CY), 67 versus 123 (9.44% versus 14.25%, ME) and 85 versus 15 (11.97% versus 1.74%, ER), respectively, see Fig. 3a. The distributions of UAGs and DAGs in subcellular locations are significantly different (*P* = 1.971e-16, chi-squared test). Noted that there is a striking difference in the fraction of UAGs and DAGs in ER, suggesting the UAG group includes far more secretory protein genes.

Next, we focused on the biological processes and pathways. GO biological process analysis was carried out for 626 UAGs and 772 DAGs mapped in DAVID. A total of 759 and 549 enriched terms were filtered for UAGs and DAGs with the threshold of *P*-value < 0.05. Most terms enriched with UAGs are relevant to the immune system, such as processes related to immune cells, ‘response to stimulus’ (54.8%, *P* = 1.10e-10) or ‘defense response’ (15.10%, *P* = 1.20e-10). In all, 178 UAGs are most significantly enriched in the term ‘immune system process’ (25.1%, *P* = 1.40e-19), whereas 292 are enriched in the term ‘cell communication’, with a fairly high number (41.1%, *P* = 1.10e-6) (Supplementary Fig. 1). Apart from processes related to cell migration, the data showed that cell proliferation, cell adhesion and cell differentiation are also highly enriched with UAGs, hinting that UAGs are likely to be involved in processes relevant to tumorigenesis.

As for DAGs, terms related to catabolic processes are enriched most significantly (with 31 terms in total); whereas metabolic process-relevant terms appear in most genes (with 80 terms in total). In total, 87 DAGs (10.1%, *P* = 5.20e-19) are most significantly enriched in the term ‘proteolysis involved in cellular protein catabolic process’, whereas 571 genes are enriched in the term ‘metabolic process’ (66.2%, *P* = 6.40e-7). In addition, 37 terms are relevant to mitochondria, and 19 terms are associated with cell cycle (111 genes are enriched in term ‘cell cycle’, *P* = 6.30e-5). Moreover, 11 terms with the keyword ‘immune’ were also observed in DAGs enriched processes, 5 terms have the keyword ‘innate immune’ and 37 DAGs are enriched in the term ‘positive regulation of innate immune response’ (3.8%, *P* = 4.80e-6) (Supplementary Fig. 2).

Kyoto Encyclopedia of Genes and Genomes (KEGG) pathway enrichment analysis revealed 26 and 32 enriched terms (with the threshold of *P* *<* 0.05) for 257 UAGs and 355 DAGs (Figs. 3b, c). Notably, apart from being enriched in pathways related to inflammation and infection, several pathways enriched with UAGs are oncogenic pathways. DAGs are enriched in foundational metabolic pathways, which is similar with the result of the enrichment analysis for biological processes. In addition, there are pathways related to neurodegenerative diseases enriched with DAGs, as a relevant study mentions^28^. Considering DAGs are downregulated across age, this result indicates that healthy old individuals may suffer from a functional decline in relevant pathways, but this result may not serve as direct evidence for the relation between healthy aging and these diseases.

### UAGs have higher SNP density and are more enriched in disease genes

**Fig. 4:**
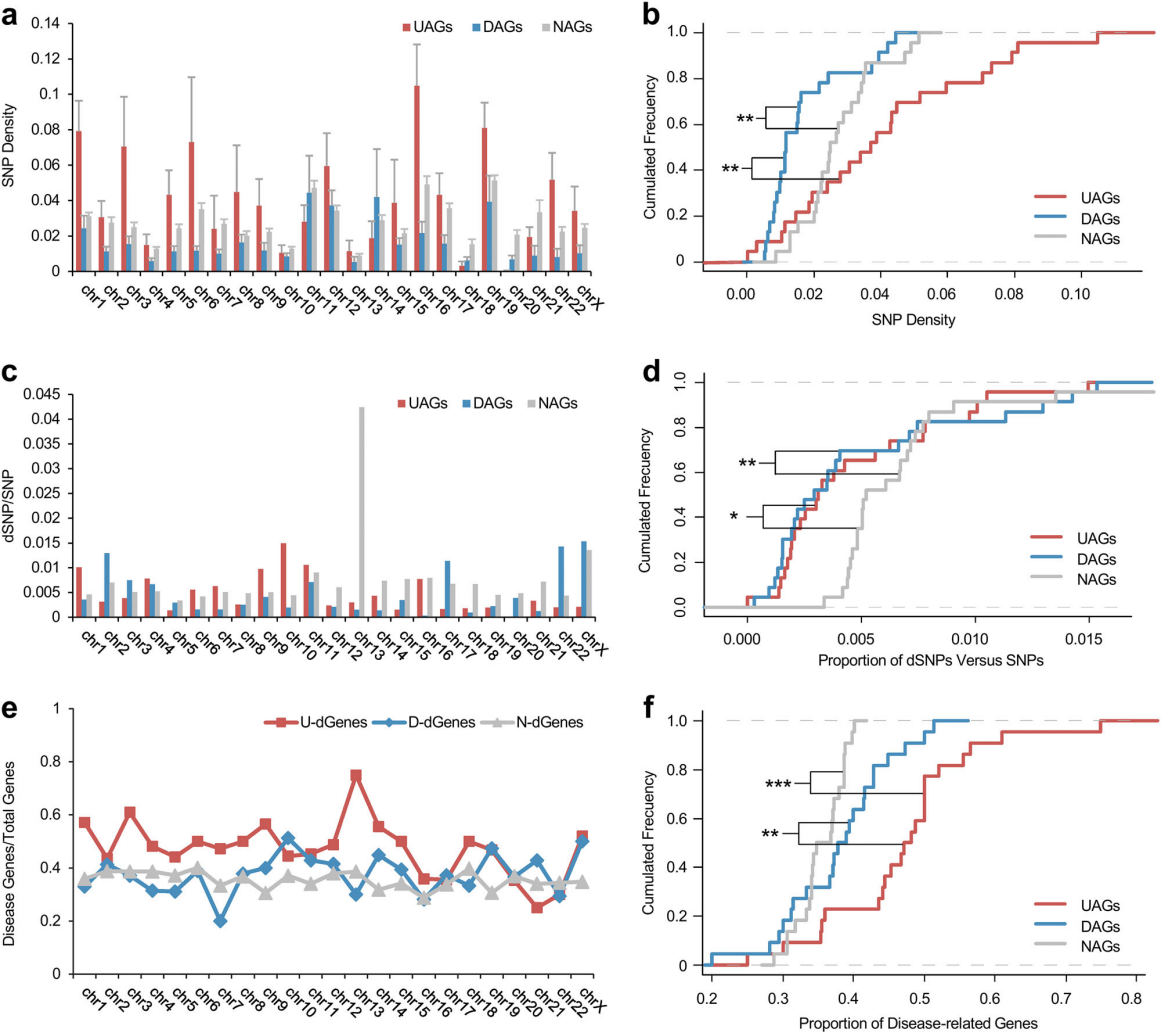
SNP distribution and disease relation of age-associated genes. **a** Distribution of average SNP density value for UAGs, DAGs and NAGs across chromosomes. The standard error of mean (SEM) in every chromosome is presented by error bars. **b** Cumulative distribution plot for the average SNP density across chromosomes. **c** Distribution for the fraction of disease-related SNP (dSNP) in the total number of SNPs across chromosomes among UAGs, DAGs and NAGs. **d** Cumulative distribution plot for the proportion of dSNP compared with total SNP. **e** The proportion of disease-related genes contained in UAGs, DAGs and NAGs across chromosomes. UAGs presented a much closer relationship with diseases. dGenes: disease-related genes; U, D, N: short for UAG, DAG, NAG, respectively. **f** Cumulative distribution plot for the proportion of disease-related genes. *P < 0.05, **P < 0.01, ***P < 0.001 from Wilcoxon test

In the human genome, single-nucleotide polymorphism (SNP) is the most common genetic variation. To investigate the difference between SNP in age-associated gene and other genes, we calculated the SNP density (the number of SNPs in a gene divided by the gene’s length) for UAGs, DAGs and NAGs, and compared their distributions across chromosomes (Fig. 4a). As expected, DAGs bear the lowest SNP density compared with UAGs and NAGs (*P* = 0.0012 and 0.0011, respectively, Wilcoxon test, the median value of UAGs, DAGs and NAGs are 0.037, 0.012 and 0.025, respectively; Fig. 4b), which is consistent with the evolutionary characteristic analysis that DAGs tend to be more conserved.

To further study whether UAGs have more disease-relevant SNPs (dSNPs) compared with other groups, we mapped the dSNPs to each gene, and calculated the fraction of the number of dSNPs, versus total number of SNPs, in each gene group (Fig. 4c). Surprisingly, no significant difference was found between the age-associated genes (data not shown), whereas NAGs have a higher dSNP fraction than UAGs and DAGs (*P* = 0.011 and 0.0045, respectively, Wilcoxon test, the median value for the fraction of dSNP to total SNP for UAGs, DAGs and NAGs is 0.31%, 0.29% and 0.52%, respectively; Fig. 4d). Given that the individuals contributing age-associated genes are healthy, it is not likely that they have many fatal mutations as reflected in the UAGs and DAGs.

In addition, 7291 disease genes from DisGeNET database were used to analyze the enrichment of the three gene groups to see whether there are differences between groups (Fig. 4e). Results showed that UAGs are enriched the most in disease genes, with the median proportion of 47.69% across chromosomes, compared with DAGs (38.36%, *P* = 0.0031, Wilcoxon test) and NAGs (35.82%, *P* = 1.02e-4, Wilcoxon test). No significant difference was observed between DAGs and NAGs (Fig. 4f). Disease genes in UAGs and DAGs are listed in Supplementary Table 4.

Moreover, for 495 UAGs, 93 items are significantly enriched in Genetic Association Database (GAD) diseases (with threshold *P* *<* 0.05). In all, 134 UAGs are most significantly enriched in term ‘Type 2 Diabetes|edema|rosiglitazone’ (18.9%, *P* = 9.30e-9), whereas ‘hypertension’, ‘multiple sclerosis’, various infectious diseases and cancers were also observed. As for the GAD disease class, 162 UAGs are significantly enriched in ‘pharmacogenomics’ (22.8%, *P* = 5.10e-10), also there are 172, 153, 106 and 182 UAGs enriched in terms ‘immune’, ‘cancer’, ‘infection’ and ‘cardiovascular’, respectively. DAGs are only enriched in one GAD item ‘infection’, with the proportion of 19.2% (*P* = 1.10e-14) (Supplementary Fig. 3).

### Age-associated genes in PPI and signaling networks

**Fig. 5:**
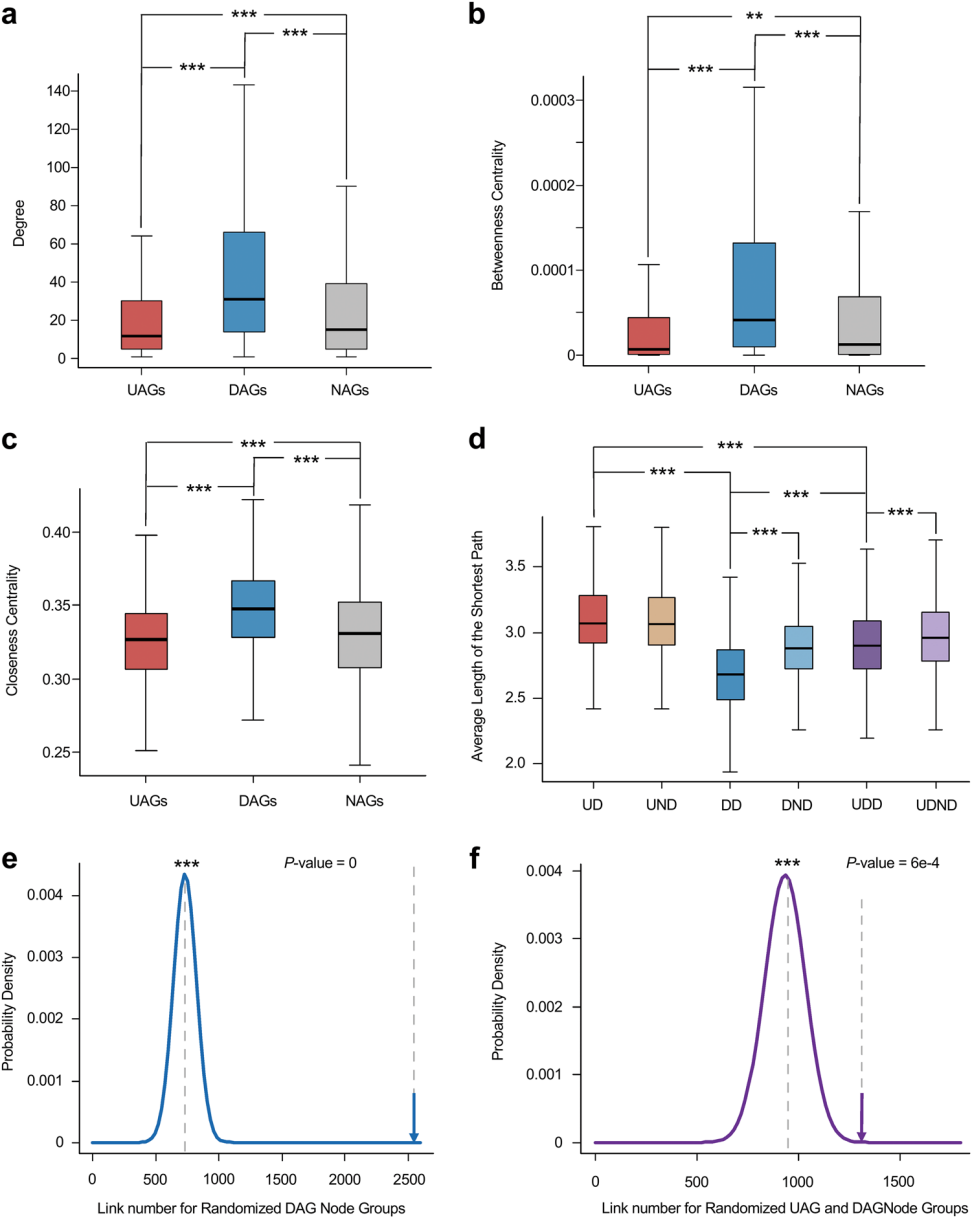
Characteristics of age-associated genes in the PPI network. **a-c**: Box plots display the difference among UAGs, DAGs and NAGs for the following characteristics in the PPI network: **a** Degree. **b** Betweenness centrality. **c** Closeness centrality. **d** Average length of the shortest paths of each group is shown in boxplot. **P *<* 0.01, ***P *<* 0.001 from Wilcoxon test. **e** The actual link number between DAG nodes, compared with link number in 10,000 randomized DAG node groups. The arrow shows link number in actual DAG node groups. **f** The actual link number between UAG and DAGs nodes compared with link number in 10,000 randomized UAG and DAG node groups. ***P *<* 0.001 from permutation test

Genes cooperate with each other to maintain homeostasis in the human body. To investigate the network characteristics of age-associated genes, we first estimated the centrality difference for each gene node in a non-directional protein–protein interaction (PPI) network through three metrics: degree, betweenness centrality and closeness centrality. These serve as important characteristics to evaluate whether a gene node in the network is ‘well-connected’ to other nodes. Among the three gene groups, DAG nodes have the highest degree, betweenness centrality and closeness centrality, compared with UAG nodes and NAG nodes, whereas UAG nodes have lower degrees than NAG nodes (Figs. 5a–c). Statistical results are shown in Table 2. To further explore how closely UAGs and DAGs are linked to each other in the PPI network, first we performed a permutation test, which revealed that the number of direct DAG-DAG links (DLs) and UAG-DAG links (UDLs) tend to be greater than when gene nodes are randomly distributed to the three gene groups (*P* = 0 and 6e-4, respectively) (Figs. 5e, f), whereas no significant results were observed for UAG-UAG links (ULs). Similar results, revealed by network distance analysis, are that DAGs have the shorter distances from each other than NAG nodes (median value of average D-D distances and D-N distances are 2.68 and 2.88, respectively), UAGs have the longest distances within the group, with no significant difference between the distance to NAGs (median value of average U-U distance and U-N distance are 3.07 and 3.06, respectively), and the UAG-DAG distances remain shorter than UAG-UAG distances (Fig. 5d and Table 3). These results suggest DAGs are of great connectivity and are cross-functioning, whereas UAGs are likely to stay in smaller groups and be more specialized in function. The fact that these two groups are fairly connected to each other in the PPI network, implies some of the UAGs and DAGs function similarly and may be attached to the same pathways.

**Table 2:** Statistical results for topological characteristic analyses in the networks

	**DAGs versus UAGs**	**DAGs versus NAGs**	**UAGs versus NAGs**
PPI network			
Degree			
Median	U: 12	D: 31	N: 15
W-value	141,360	7,002,200	3,693,300
* P*-value	4.30e-36	1.35e-45	2.99e-4
Betweenness centrality			
Median	U: 7.26e-06	D: 4.13e-05	N: 1.23e-05
W-value	148,890	6,878,900	378,200
* P*-value	1.11e-30	3.84e-39	0.0011
Closeness centrality			
Median	U: 0.327	D: 0.347	N: 0.331
W-value	138,520	7,041,200	3,693,600
* P*-value	3.11e-38	1.10e-47	3.06e-4
Signaling network			
Positive regulation links in out-degree			
Proportion	U: 80.79% (2057 of 2546)	D: 78.32% (2366 of 3021)	
Chi-square	5.03		
* P*-value	0.025		
Positive regulation links in in-degree			
Proportion	U: 78.93% (2338 of 2692)	D: 84.68% (2134 of 2520)	
Chi-square	29.57		
* P*-value	5.41e-8		
Betweenness centrality			
Median	U: 8.16e-06	D: 5.84e-07	N: 4.57e-07
W-value	52,407	887,190	914,810
* P*-value	0.027	0.75	6.15e-04
Closeness centrality			
Median	U: 0.184	D: 0.176	N: 0.174
W-value	54,246	883,440	943,580
* P*-value	0.0031	0.86	1.84e-05

The degree for PPI network, betweenness and centrality in both networks were compared by Wilcoxon test, the proportion of positive links in in-degree and out-degree in signaling network were compared by chi-squared test

**Table 3:** Statistical results for network distance analyses

	UD versus DD	UD versus UND	DD versus UDD	DD versus DND	UD versus UDD	UDD versus UDND
PPI network						
Median	UU: 3.07	UN: 3.06	DD: 2.68	DN: 2.88	UD: 2.90	UDN: 2.96
W-value	426,260	192,850	140,320	194,510	255,200	388,610
* P*-value	4.47e-117	0.423	2.11e-45	8.79e-47	1.78e-27	7.04e-5
Signaling network						
Median	UU: 3.57	UN: 3.75	DD: 3.74	DN: 3.81	UD: 3.7	UDN: 3.77
W-value	22,554	406,120	54,985	455,310	44,784	1,804,700
* P*-value	0.0040	2.19e-05	0.62	0.046	0.0021	0.018

The network distances were represented by average length of the shortest paths for nodes in each group and compared by Wilcoxon test. For the signaling network, distances were counted from both directions in each distance group

*UD* network distances between UAG nodes within the group*, DD* distances between DAG nodes within the group, *UDD* distances between UAG and DAG node groups, *UND* distances between UAG and NAG node groups, *DND* distances between DAG and NAG node groups, *UDND* distances in UND and DND were added up to UDND group

**Fig. 6:**
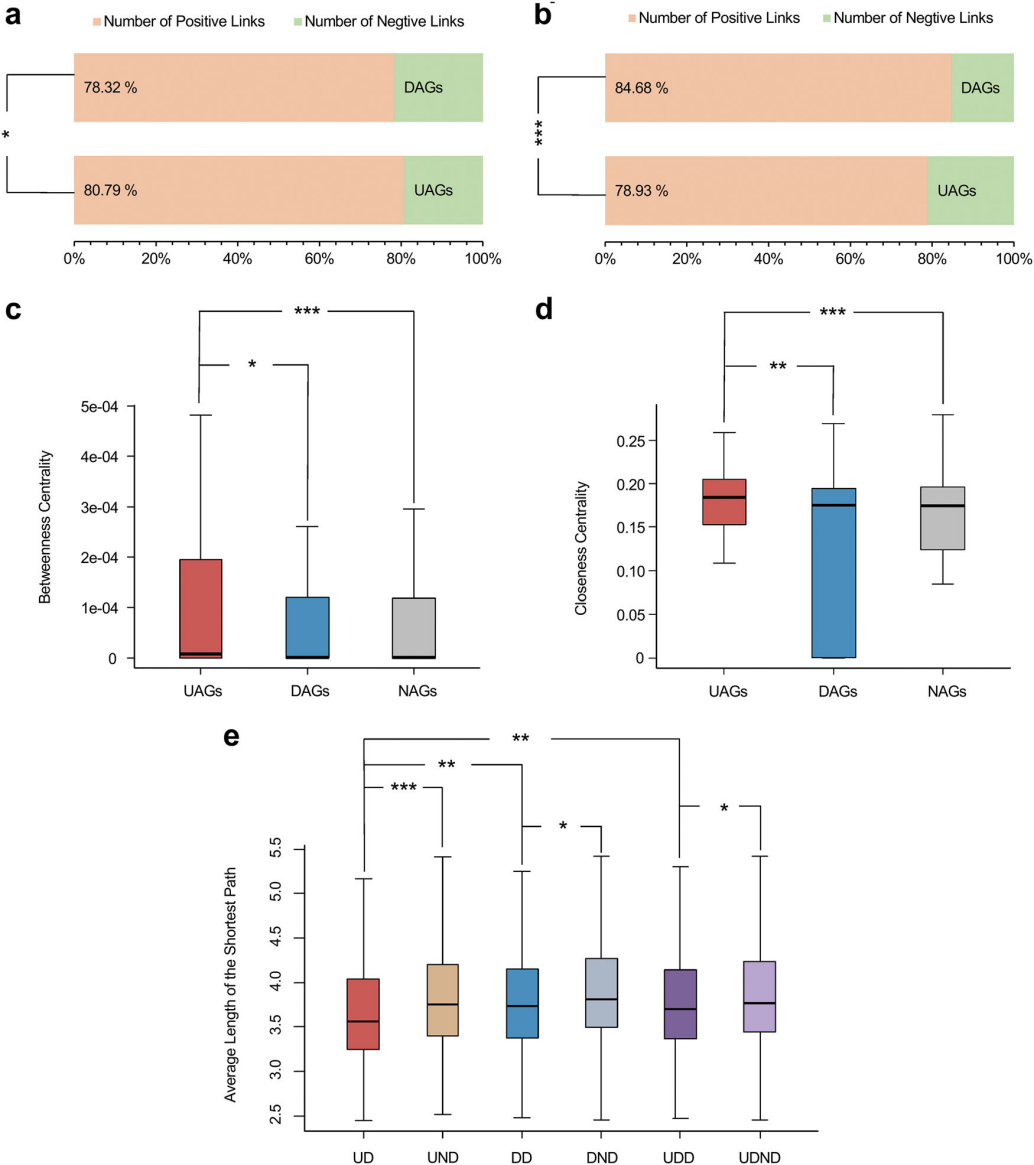
The signaling network provide more detailed characteristics for age-associated genes. **a** The proportion of positive and negative links in out-degree. UAGs have greater number of positive links than DAGs. **b** Greater number of negative links in in-degree of UAGs as compared with DAGs. *P < 0.05, ***P < 0.001 from chi-squared test. **c, d** Box plots present the difference in **c** betweenness centrality and **d** closeness centrality between groups in signaling networks. **e** The network distances revealed by average of the shortest paths are shown in boxplot. *P < 0.05, **P < 0.01, ***P < 0.001 from Wilcoxon test

In addition, we explored the characteristics of UAGs and DAGs in a cellular signaling network, in which 299 UAGs, 319 DAGs were mapped, with 5506 other genes considered as NAG nodes^29^. Centrality and distance analysis for UAGs and DAGs, in the signaling network, revealed different results, compared to PPI network, which UAGs have higher betweenness and closeness centrality value than DAGs and NAGs (Figs. 6c, d and Table 2). Concomitantly, DAGs have higher values than NAGs. Besides, UAGs have more positive links and negative links, compared with DAGs, respectively in out-degree and in-degree (Figs. 6a, b and Table 2). As for the distance analysis, more U-U links, D-D links and U-D/D-U links were observed in the UAG and DAG node groups compared with randomized groups (*P* = 0, 0, 0.021, respectively, permutation test) (Supplementary Fig. 4), whereas U-U distances remain the shortest when compared with U-D, D-D and U-N. D-D and U-D distances are also shorter, in comparison with distances to other nodes (Fig. 6e and Table 3). These results showed UAGs and DAGs both have high connectivity in the signaling network; nonetheless UAGs are more clustered and of great centrality, suggesting that UAGs are more interconnected in the signaling network.

## Discussion

Aging is a process characterized by progressive loss of physiological integrity, which leads to impaired function and increased vulnerability to death^30^. By analyzing the aging differences revealed by gene expression, important clues can be gained to better understand the process of aging at the transcriptional level. Here, we systematically characterized the characteristics of transcriptional age-associated genes in multiple aspects. To sum up, genes that increase expression with age are less conserved in evolution, more tissue specific in expression and more enriched in SNPs and disease genes. They are more active in the signaling network and are located more in the extracellular region, with clustered functions mostly involving the immune system and are likely to play crucial roles in various cancer-related pathways. Genes with a decreased level of expression with age have a longer phyletic history, tend to be stable over time, expressed globally across tissues and are expressed early in a human lifetime. These genes function mostly in basic metabolic or catabolic process that are vital for human survival, and play important roles in gene networks, especially in the PPI network.

Notably, these age-associated genes, in the up and downregulated directions, tend to be close to each other in genomic distances and distances in both PPI and signaling networks. To further study potentially aging-related processes, 323 interacting UAGs and DAGs nodes were extracted from the signaling network, visualized (Supplementary Fig. 5) and functionally annotated (results for GO BP and pathway enrichment analysis with threshold FDR < 0.05 are shown in Supplementary Tables 5 and 6, respectively) by Cytoscape^31^. Examples for the interacting age-associated gene nodes enriched in process ‘T-cell receptor signaling pathway’ and enriched in pathway ‘pathways in cancers’ are shown in Supplementary Figure 6.

Apart from this, some DAGs are overlapped in function with UAGs, other DAGs are more dispersive with functions in multiple aspects, whereas UAGs are consistently more clustered and co-functioned. These dispersive distributed DAGs are enriched in terms related to catabolic, metabolic, mitochondrion-related and innate immune processes, which is consistent with previous studies that aging is a process with mitochondrial and immune dysfunction^9,32^. UAGs are mostly clustered in immune-related function and more located in the extracellular region, indicating the tendency of senescent cells to secrete pro-inflammatory cytokines, which lead to a chronic inflammatory state in healthy aging individuals. Besides, these immune-relevant UAGs and some of their interacted DAGs are also highly enriched in oncogenic signaling pathways, including Ras, nuclear factor kappa-light-chain-enhancer of activated B cells(NF-kappa B), phosphatidylinositol 3-kinase(PI3K)/AKT, Notch and P53 signaling pathways, which implies that senescent cells resemble cancer cells. This needs to be further verified by comparing aging profiles to specifically precancerous profiles. When mapping the 1020 cancer genes from OncoKB^33^ to all gene groups, UAGs were observed to bear the highest cancer genes proportion (7.75%) compared with DAGs (4.17%, *P* = 0.0036, chi-squared test) and NAGs (4.97%, *P* = 0.0013, chi-squared test). DAGs have the lowest proportion and no significant difference is shown between DAGs and NAGs. The cancer genes mapped in UAGs and DAGs are listed in Supplementary Table 7. In addition, we observed 15 and 14 cancer driver genes, respectively, from a 299 pan-cancer driver gene set provided by a recent study of TCGA’s Pan-Cancer^34^ in UAGs and DAGs, such as *ERBB2* (level 1 driver gene in OncoKB^33^), *TNFAIP*3 in UAGs, and *MAPK1*, *PIK3CB* in DAGs (driver genes mapped in UAGs and DAGs are listed in Supplementary Table 8). As these driver genes are mostly increased in expression in tumorigenesis process, the driver genes found in DAGs may serve as a protective factor in this driving process. However, a previous study showed some driving processes are not likely to co-occur in cancer^35^, therefore the decreased level in these driver genes may accompany an increased level of other driver genes.

Aging is widely considered to be caused by accumulated cellular damage^36,37^, which is led by random mutations. SNP enrichment analysis shows more variations in UAGs, which means these genes are likely to be the positions that random mutation occurred. There is a possibility that the mutation in some UAGs cause them to increase in expression, and interfere with other interactive DAGs and UAGs. These changes may contribute to part of the aging transcriptional landscape. Considering DAGs experience less selection with lower evolutionary rates, mutations in DAGs are likely to be fatal, which would cause more disruptive outcomes that would be hard to observe in healthy individuals. Besides, as previous studies noted^13,38,39^, the decreased expression in DAGs may be mainly caused by age-associated epigenetic drift. The analyses in evolutionary characteristics, expression pattern and connectivity in the PPI network, showed consistent results with previous studies that UAGs are younger genes than DAGs, as studies showed that younger genes evolve more rapidly^40^, are more likely to present different temporal and spatial expression patterns^41^ and have fewer interactions in the PPI network^42^. Although UAGs and DAGs are both of high connectivity in the signaling network, UAGs remain more interactive, which suggests aging may accompany more accumulated in signaling crucial proteins.

Moreover, when performing the functional enrichment analysis, it was of note that the number of enriched terms that have a positive and a negative regulating function are about the same. This suggests those differentially expressed genes in healthy old individuals are not likely to cause imbalances that lead to conspicuously disruptive outcomes. As for an effort in precise medicine, 82 and 88 FDA-approved target genes from DrugBank^43^, mapped in UAGs and DAGs (Supplementary Table 9), are provided, respectively, which hopefully can help future research in different drug use for the young and old. Overall, our findings provide multiple biological implications for further study in healthy aging.

## Materials and methods

### The age-associated gene set

The age-associated gene set was extracted from a dataset of genes expressed differentially across age, as given in the GTEx project. These genes were screened out using a linear mixed model where sex, race and tissue were controlled to avoid the biases^24^. Our study focused on protein-coding genes, thus noncoding RNA genes were excluded from the gene set and the remaining genes were then categorized into two groups according to the regression coefficient. Genes with positive and negative coefficients were described as UAGs and DAGs, respectively. The original Ensembl IDs in the dataset were transformed into Entrez gene IDs and official gene symbols for further analysis. Apart from the age-associated genes, the rest of the protein-coding genes were regarded as NAGs.

### Chromosomal distribution and genomic distance analyses

Chromosomal distribution of age-associated genes was revealed by calculating and comparing the proportion of both UAGs and DAGs in the totality of genes on each chromosome. The genomic distances of every pair of UAGs/DAGs (UDs/DDs), as well as the distances between a UAG and a DAG (UDDs) on the same chromosome, were calculated. The Wilcoxon rank-sum test was used to compare the distribution of UDs, DDs and UDDs across chromosomes.

### Evolutionary and expression characteristic analyses

The average expression level across tissues and tissue specificity of each gene were calculated based on a gene atlas database from Su et al.^44^. The dN/dS ratio dataset of each human–mouse homolog was derived from the Ensembl database (release 83) to illustrate gene evolutionary rate. The homologous gene number dataset was obtained from the Homologene database^45^ (build 68), the phyletic evolution and earliest expression stage datasets were obtained from Online Gene Essentiality database^46^ and the essential gene dataset was obtained from the DEG database (version 10.6)^27^. The Wilcoxon test was performed to statistically compare the evolutionary rate, expression level, expression specificity and homologous gene number of UAGs, DAGs and NAGs, whereas a chi-squared test was used to compare the proportion of earliest expression stage, phyletic evolution and the essential genes between the three gene sets.

### Functional enrichment analysis for age-associated genes

We calculated the number of genes in four terms of the GO: NU, CY, ME and ER, and compared the numbers between UAGs, DAGs and NAGs by performing chi-squared tests. Meanwhile, functional enrichment analysis of GO biological process and KEGG pathway annotations were both performed using the DAVID Web server^47^.

### Analysis of SNP densities

A dataset of SNPs in human protein-coding genes (SNPs and indels, excluding flagged variants) (GRCh38.p2) with genome coordinates was derived from the Ensembl database^48^. SNP density of each gene was defined as the total number of mapped SNPs, divided by gene length. SNPs were mapped to all protein-coding genes, and the average SNP densities on each chromosome of UAGs, DAGs and NAGs were compared using the Wilcoxon test.

### The enrichment analysis of disease-related SNP and disease genes

The disease-related SNPs (dSNPs) were obtained from the ClinVar database^49^ and Human Gene Mutation Database;^50^ data without a dbSNP ID or labeled as ‘protective’, ‘(likely) benign’, ‘uncertain significance’, ‘conflicting data from submitters’, ‘other’ and ‘not provided’ were excluded. dSNPs were mapped to each chromosome and the fractions of dSNPs to total SNPs in UAGs, DAGs and NAGs were calculated and compared using the Wilcoxon test. As for disease gene analysis, the DAVID Web server^47^ was used to perform functional enrichment analysis for terms of specific disease and disease class in the GAD, and the curated gene–disease association dataset downloaded from the DisGeNET database^51^ was also used to compare the fraction of disease genes for UAGs, DAGs and NAGs across chromosomes by performing the Wilcoxon test.

### Network analysis

The human PPI network was downloaded from the BioGRID database (build 3.4.140) with the deletion of links that include non-human protein^52^. The human cellular signaling network was taken from our previous study^29^. The Wilcoxon test was performed to investigate the degree, betweenness centrality and closeness centrality differences for the UAG, DAG and NAG nodes in the PPI network. For the degree analysis in the signaling network, the proportion of positive links to the sum of positive links and negative links in UAGs and DAGs was compared for both in-degree and out-degree by performing chi-squared tests. As for network distance analysis, permutation tests were performed to compare the numbers of UAG-UAG links (ULs), DAG-DAG links (DLs) and UAG-DAG/DAG-UAG links (UDLs) in our identified networks with the numbers of those three types of links for 10,000 randomized node groups. The *P*-value was calculated as the frequency of times when link number in randomized groups was greater than that in identified groups. In addition, the average length of the shortest paths for UAG and DAG nodes in their own group, between each group and with the NAG nodes were also compared by performing the Wilcoxon test. The degree, betweenness centrality, closeness centrality and length of the shortest path values were calculated by using the python package NetworkX^53^. For the signaling network, distances were counted from both directions in each distance group. Network visualization and functional enrichment analysis for interacting age-associated gene nodes in the signaling network were carried out by Cytoscape^31^.

## Electronic supplementary material

Supplementary Figures 1–6

Supplementary Tables 1–9
