# Supplementary material for: An analysis of aging-related genes derived from the Genotype-Tissue Expression project (GTEx)
Source: Cell Death Discov. 2018 Aug 20;4:91. doi: 10.1038/s41420-018-0093-y (PMC6102484; doi:10.1038/s41420-018-0093-y)
Supplement: Supplementary file 1 — Supplementary Figures 1–6 [file 41420_2018_93_MOESM1_ESM.docx]

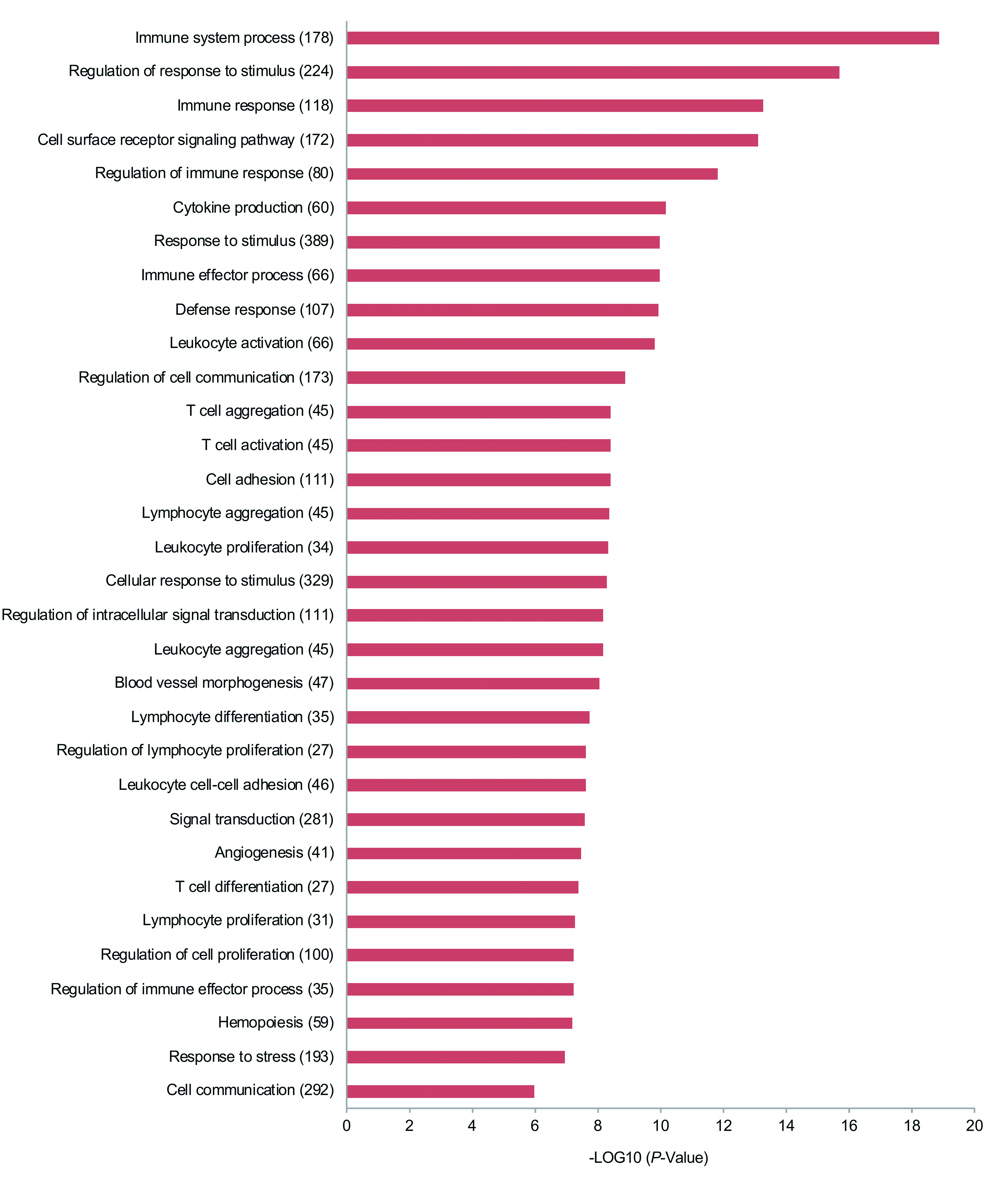


**Supplementary Figure 1. GO BP enrichment analysis for UAGs.** Part of the GO BP enrichment result for UAGs is shown.


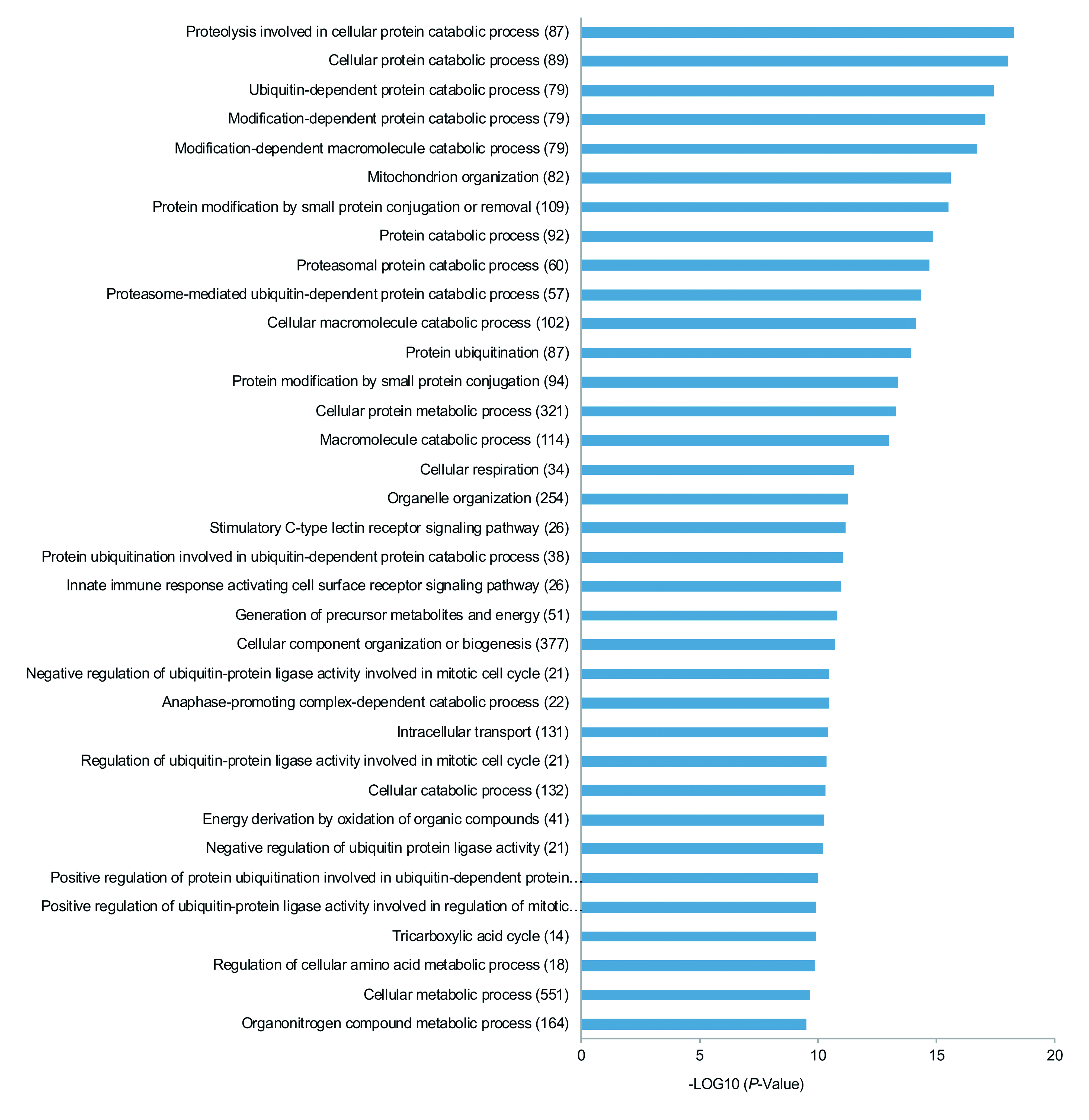


**Supplementary Figure 2. GO BP enrichment analysis for DAGs.** Part of the GO BP enrichment result for DAGs presented in the bar graph.

**
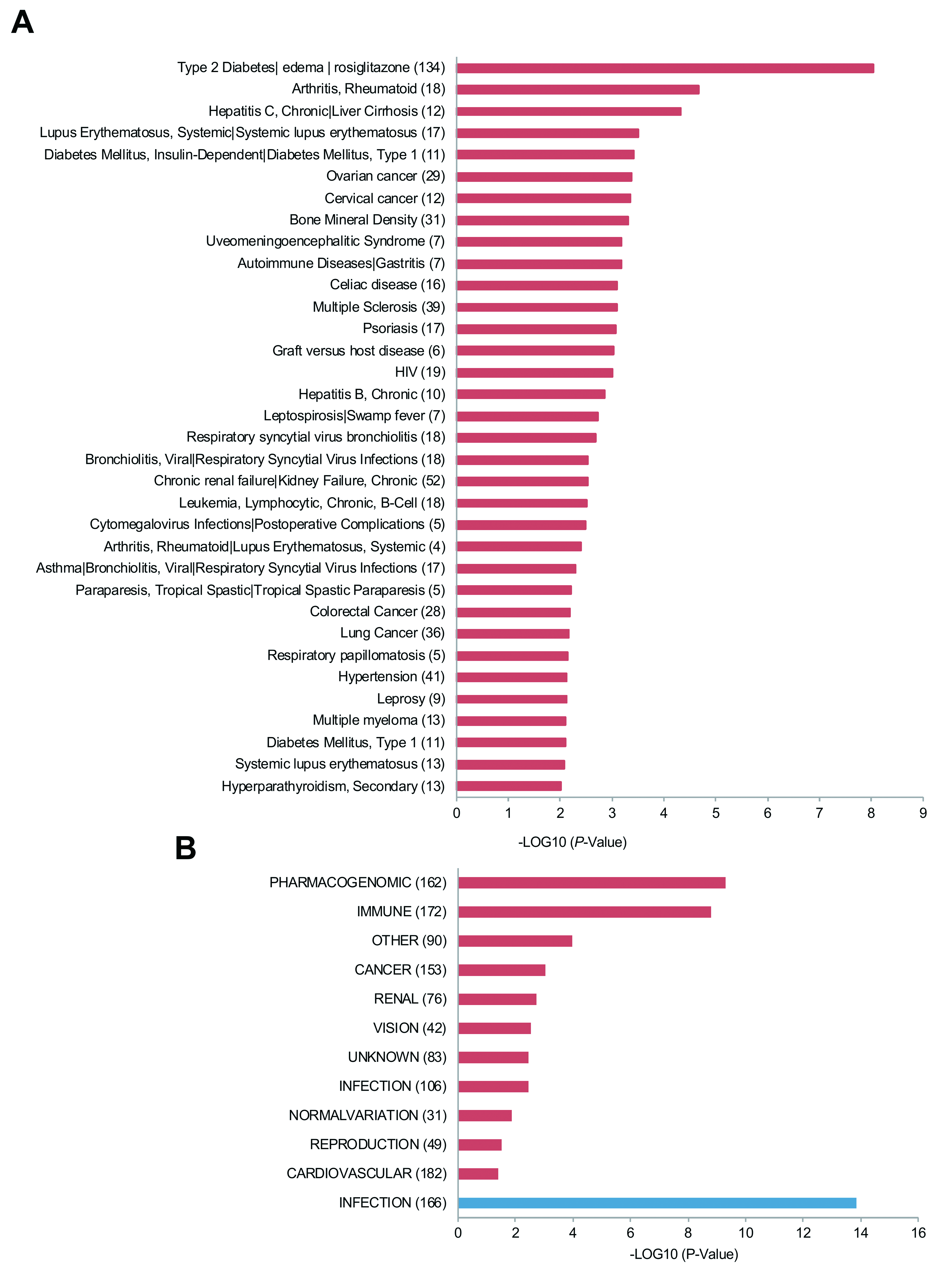
**

**Supplementary Figure 3. GAD disease enrichment analysis.** (A) The Bar graph displays the GAD disease enrichment result for UAGs with the threshold of *P* <0.01. (B) The GAD disease class enrichment result is shown with the threshold of *P* <0.05. Red bars represent UAGs while blue bars represent DAGs.

**
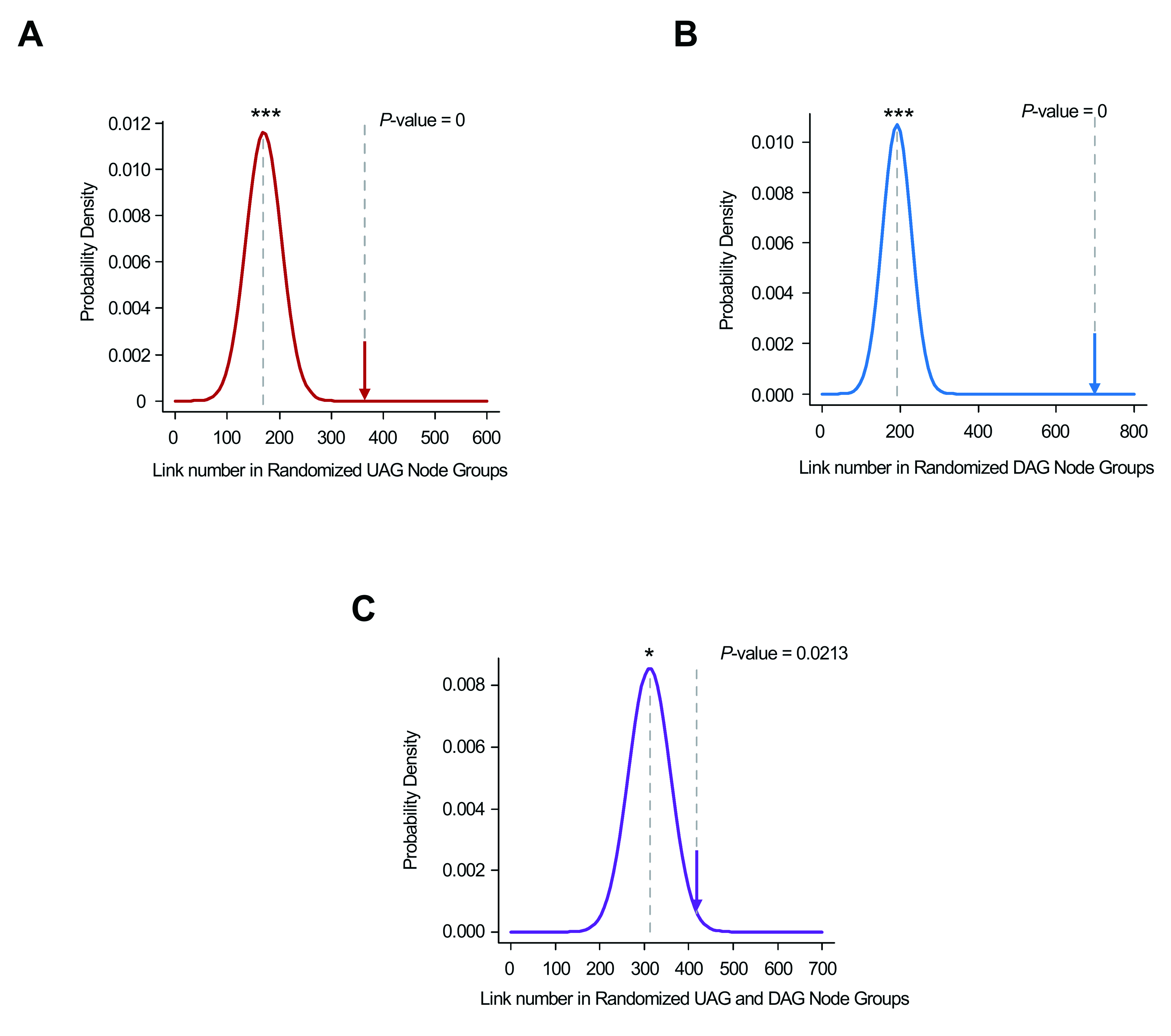
**

**Supplementary Figure 4. The age-associated genes tend to interact with each other in the signaling network.** Frequency for greater number in randomized node groups compared with link number in actual node groups for (A) UAG-UAG links; (B) DAG-DAG links; and (C) UAG-DAG links. The low frequencies indicate that age-associated genes tend to be more interactive with each other in the signaling network.

**
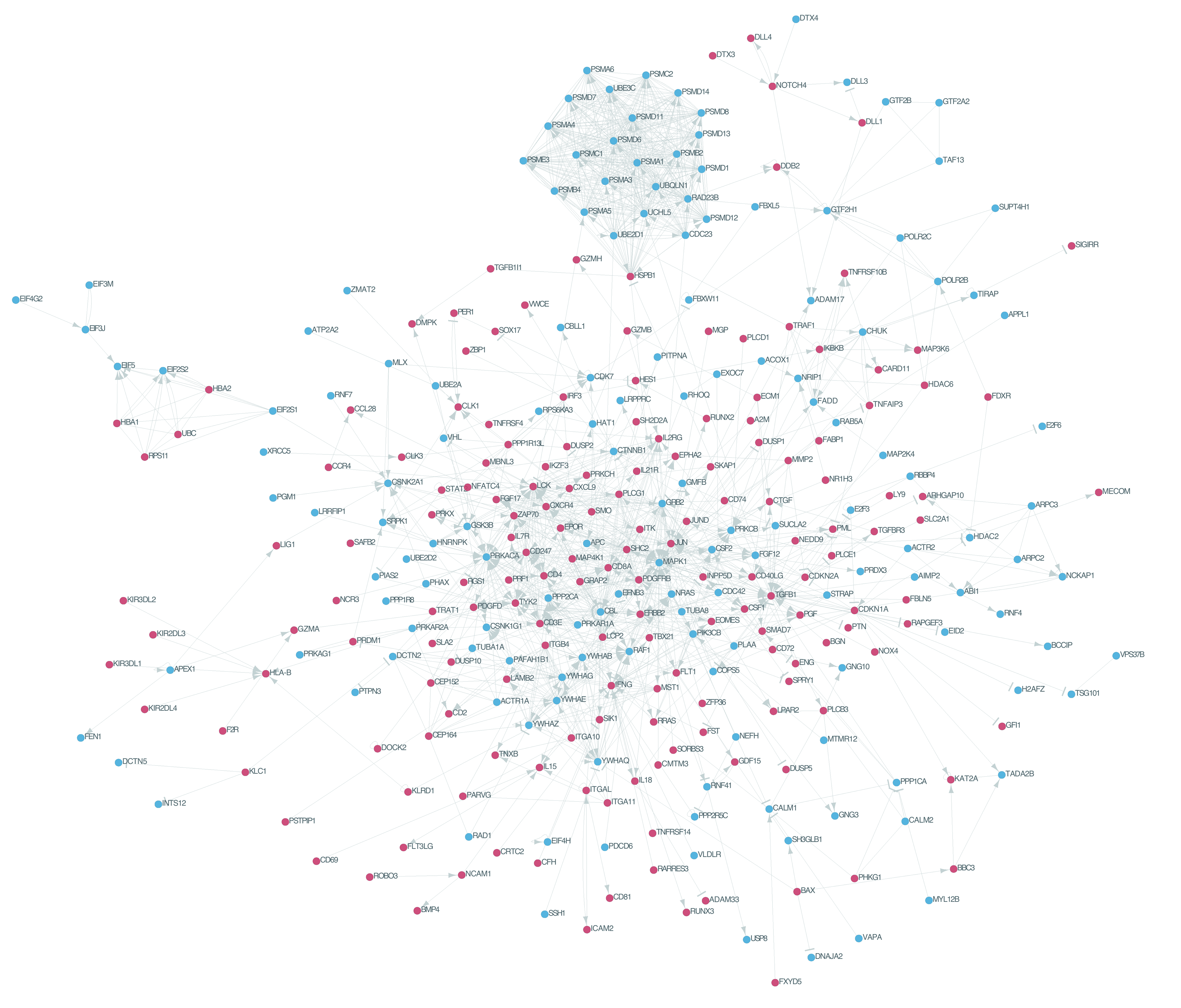
**

**Supplementary Figure 5. The interacting age-associated genes in the signaling network.** The 323 interacting age-associated gene nodes is displayed, with 172 UAG (red) and 151 DAG (blue) nodes. Edges in arrow, T-shaped and full line represent positive, negative and physical interactions, respectively.

**
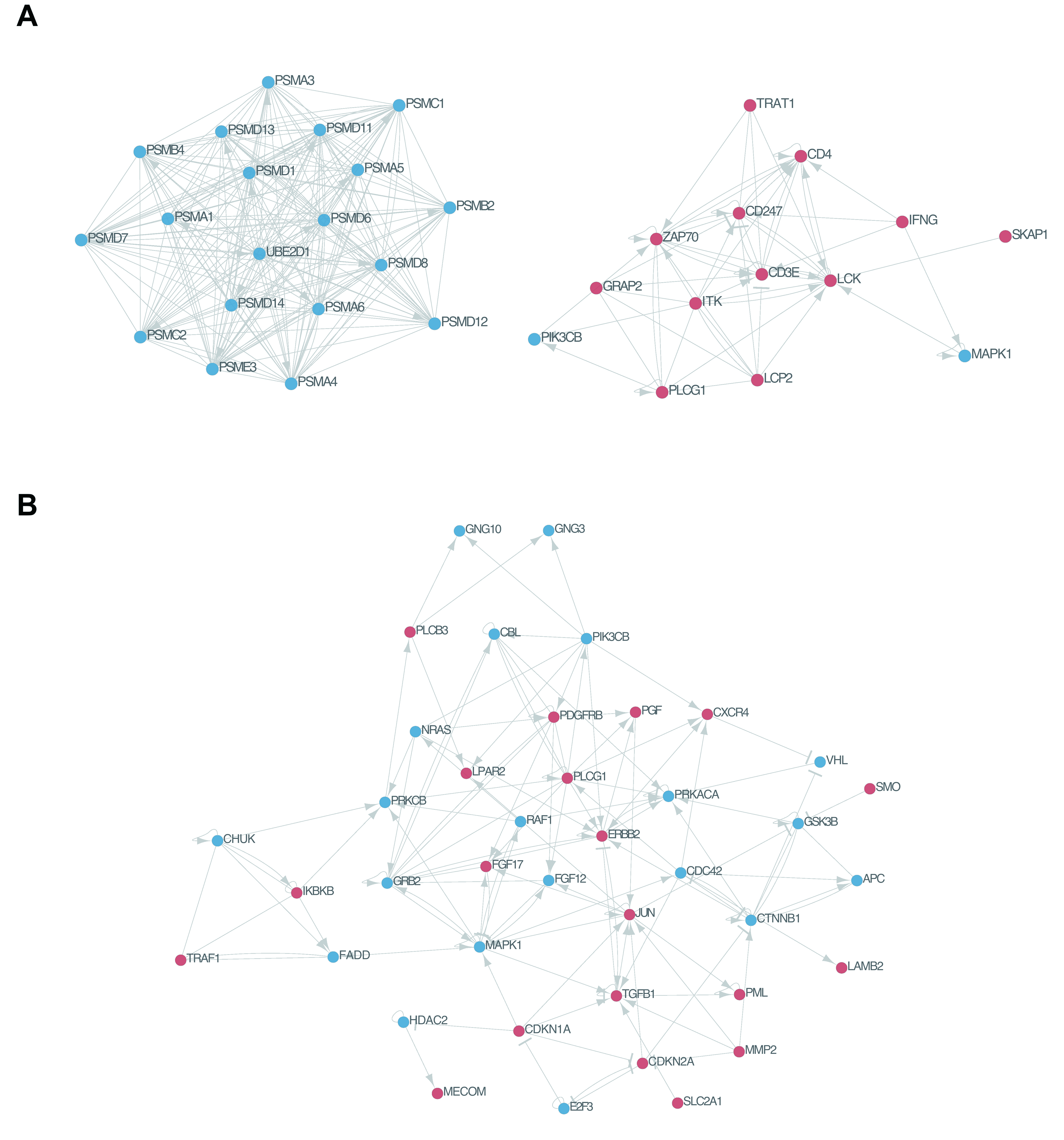
**

**Supplementary Figure 6. Examples for GO BP and pathway enrichment analysis for the interacting age-associated gene nodes in signaling networks.** UAG are presented in red, and DAG nodes are presented in blue. Edges in arrow, T-shaped and full line represent positive, negative and physical interactions, respectively. (A) 33 interacting gene nodes enriched in process ‘T cell receptor signaling pathway’ (B) 40 interacting gene nodes enriched in KEGG pathway ‘pathways in cancer’.
